# Anxiety symptoms in critically ill COVID-19 survivors and its association with post-discharge health concerns

**DOI:** 10.1192/j.eurpsy.2022.1368

**Published:** 2022-09-01

**Authors:** B. Silva, S. Martins, A.R. Ferreira, J. Fernandes, T. Vieira, L. Fontes, N. Reis, A. Braga, I. Coimbra, J.A. Paiva, L. Fernandes

**Affiliations:** 1 Faculty of Medicine University of Porto (FMUP), Fmup, Porto, Portugal; 2 Faculty of Medicine - University Porto, Department Of Clinical Neuroscience And Mental Health And Center for health technology and services research (cintesis), Porto, Portugal; 3 Centro Hospitalar Universitário São João (CHUSJ), Intensive Care Medicine Department, Porto, Portugal; 4 Faculty of Medicine - University Porto, Department Of Medicine, Porto, Portugal; 5 Psychiatry Service, Centro Hospitalar Universitário De São João, Porto, Portugal

**Keywords:** Covid-19, Anxiety, Quality-of-life, Critical illness

## Abstract

**Introduction:**

Evidence suggest that critically ill COVID-19 patients are at higher risk of developing anxiety symptoms, which may be related to or exacerbated by patients concerns regarding their health status and recovery.

**Objectives:**

To assess anxiety symptoms in critically ill COVID-19 survivors, 1-2 months after hospital discharge and to analyze its association with concerns reported by patients regarding their own health status and recovery.

**Methods:**

In the framework of MAPA prospective research, this preliminary study included COVID-19 patients admitted in the Intensive Care Medicine Department (ICMD) of a University Hospital. Patients were excluded if they had an ICMD length of stay (LoS) ≤24h, terminal illness, major auditory impairment or inability to communicate at the evaluation time. Participants were assessed at a scheduled telephone follow-up appointment, with Generalized Anxiety Disorder Scale (GAD-7). Additional questions were asked to assess the survivors’ post-discharge concerns regarding discrimination against for COVID-19, infection of a family member, re-infection or sequelae related to COVID-19.

**Results:**

Eighty-three patients were included (median age=63 years; 63% male) and 24% had anxiety symptoms. Anxiety scores were higher in survivors who reported being afraid of being discriminated against for COVID-19 (30% vs 10%; p=0.034), being re-infected (100% vs 79%; p=0.032) and having sequelae (94% vs 44%; p<0.001).

**Conclusions:**

These findings revealed that anxiety is common in COVID-19 survivors and is associated with post-discharge patients concerns that may limit patient daily living. This study emphasizes the importance of psychological assessment and follow-up of the COVID-19 survivors, in order to support these patients recovery.

**Disclosure:**

No significant relationships.

